# Radiofrequency treatment alters cancer cell phenotype

**DOI:** 10.1038/srep12083

**Published:** 2015-07-13

**Authors:** Matthew J. Ware, Sophia Tinger, Kevin L. Colbert, Stuart J. Corr, Paul Rees, Nadezhda Koshkina, Steven Curley, H. D. Summers, Biana Godin

**Affiliations:** 1 Department of Nanomedicine, Houston Methodist Research Institute, Houston, Texas, USA; 2 Centre for Nanohealth, College of Engineering, Swansea University, Swansea, UK; 3 Baylor College of Medicine, Houston, Texas, USA; 4 MD Anderson Cancer Centre, Houston, Texas, USA

## Abstract

The importance of evaluating physical cues in cancer research is gradually being realized. Assessment of cancer cell physical appearance, or phenotype, may provide information on changes in cellular behavior, including migratory or communicative changes. These characteristics are intrinsically different between malignant and non-malignant cells and change in response to therapy or in the progression of the disease. Here, we report that pancreatic cancer cell phenotype was altered in response to a physical method for cancer therapy, a non-invasive radiofrequency (RF) treatment, which is currently being developed for human trials. We provide a battery of tests to explore these phenotype characteristics. Our data show that cell topography, morphology, motility, adhesion and division change as a result of the treatment. These may have consequences for tissue architecture, for diffusion of anti-cancer therapeutics and cancer cell susceptibility within the tumor. Clear phenotypical differences were observed between cancerous and normal cells in both their untreated states and in their response to RF therapy. We also report, for the first time, a transfer of microsized particles through tunneling nanotubes, which were produced by cancer cells in response to RF therapy. Additionally, we provide evidence that various sub-populations of cancer cells heterogeneously respond to RF treatment.

Cellular phenotype is the conglomerate of multiple cellular processes involving gene and protein expression that result in the elaboration of a cell’s particular morphology and function^1^. Changes in cell phenotype are usually a consequence of an adaptive behavior to micro/macro environmental stimuli. As an example, in the case of certain cells these changes can point towards alterations in invasiveness^2^. Hence, physical cues in the mechanistic study of cancer are gaining more and more attention in recent years, as their importance is gradually being realized. These measurements provide 1) information on any changes in cellular behavior, such as migratory or communicative changes, in response to a specific treatment or as a result of the progression of the disease^2^, and 2) insight into intrinsic differences in the physical properties of malignant cells verses their non-malignant counterparts. Radiofrequency (RF) is one of the methods used to treat tumors^3^,^4^. Currently, only invasive RF techniques are applied in the clinic, which is based on surgically exposing the tissue of interest to heat generated from high frequency alternating current aiming to ablate the tumor and surrounding healthy tissue^5^. Non-invasive RF therapy^3^,^6^,^7^ is a promising way to treat virtually any type of tumor and is about to be clinically tested in the next few years. This technique uses externally applied radio-waves which possess a low specific absorption rate in living healthy tissues^7^. The proposed mechanism by which tumor tissue is being eliminated is based on an impaired blood flow in the tumor^8^ and, hence heat dissipation^9^,^10^. Thus, cancer cells could be destroyed or induced into apoptosis while leaving healthy tissue relatively unharmed. However, effects of non-invasive RF on the physical features, or cellular phenotype, of single cancer and non-cancerous cells have not been fully elucidated.

Here we report the physical responses of two pancreatic cancer cell lines (AsPc-1, and PANC-1) and one normal pancreatic cell line (HPDE) after single and multiple RF treatments. Cells were evaluated with a battery of physical measurements, as outlined in Table 1. These measurements encompass observations on multiple lengths scales including molecular, subcellular, cellular and population wide length scales, as biological functions and behaviors result from complex mechanisms which occur cross diverse scales^11^. Where possible we used high-throughput analysis of the same cell population before and RF treatment to achieve observations that represent the response of a single cell population, as population susceptibility differences to RF may skew the results obtained. Furthermore, high throughput analysis possesses many benefits^12^, which include the achievement of statistically robust findings. The measurement of phenotypic differences in pancreatic cancer cell lines can provide mechanistic insights through linkage of differential expression of specific proteins to tumor growth, invasion and metastasis^13^,^14^ and chemotherapeutic drug response and resistance^15^. This is particularly important, as currently there is a limited understanding regarding the alteration in pancreatic cancer cell phenotype due to RF treatment or whether certain phenotypes within the heterogeneous cancer cell population respond differently to treatment than others.

## Results

### Morphology

**Figure 1:**
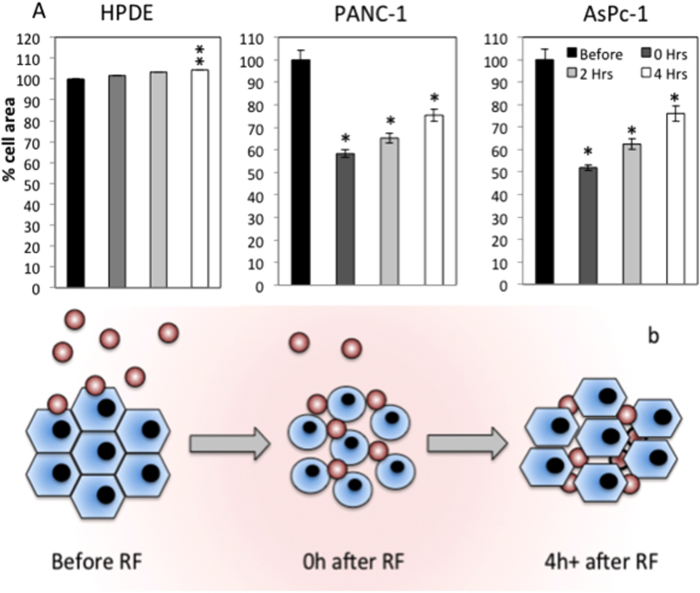
Whole cell population analysis 2-D Cell area. **a**) % cell area (normalized to the cell area before RF) at time points before, 0, 2 and 4 h after RF (n > 600 cells per group). **b**) Schematic diagram representing increased diffusion of dose (red dots) through cell monolayer after RF treatment (*p < 0.01, **p < 0.05).

**Figure 2:**
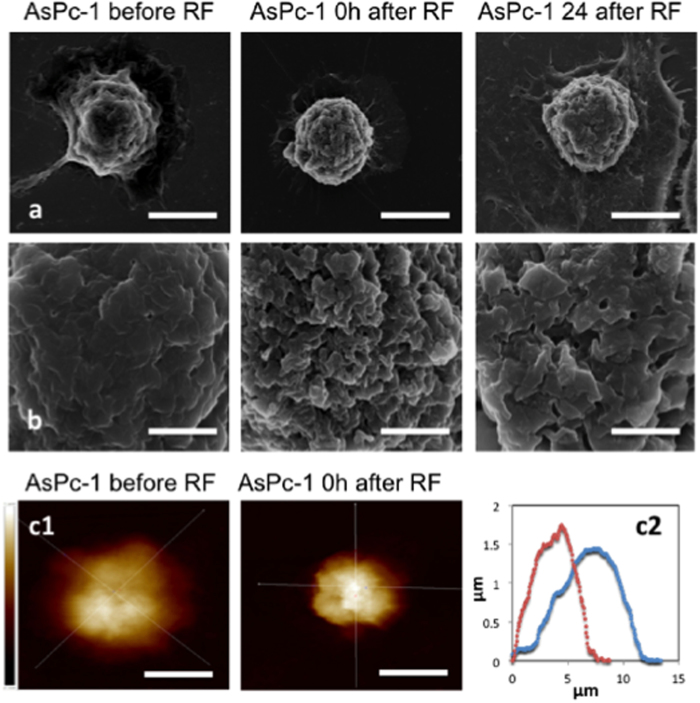
Cell level analysis of RF response. Cell hyperthermal shock indicated by cytoplasm retraction and recovery. **a** and **b**) Scanning electron micrographs of AsPc-1 cells at times points before, 0 and 24 h after RF exposure (top row 10027x magnification, scale bar 10 μm, second row from top 45293X magnification, scale bar 2 μm), **c1**) AFM topographical analysis of a single AsPc-1 representative cell before and at 0 h after RF (scale bar 10 μm and intensity scale bar: black shade and white shade represents 0 and 2.1 μm above substrate respectively and **c2**) Topography of single representative cells before RF exposure (blue line) and after RF exposure (red line). See Supplementary Fig. S3: shows SEM images of PANC-1 and HPDE at the same time points.

Morphological and size parameters of PANC-1, AsPc-1 and HPDE were characterized before and after RF. The brightfield time-lapse data showed PANC-1 and AsPc-1 cells immediately retract their cytoplasm in response to a single RF treatment (Fig. 1, Fig. S1, Supplementary). This suggested the malignant cells had undergone a form of hyperthermal shock (Fig. S2, Supplementary). Non-malignant HPDE cells when subjected to a single RF treatment did not display any significant cytoplasmic retraction or detachment from the substrate surface. Between 0 and 4 h after RF, malignant cells recovered their adhesion to the substrate surface, indicated by their recovery in size, (Fig. 1) and at 24 h full recovery of cell area had occurred. These time-resolved images of a live cell population provide details of the timing of the cellular responses to RF. This will aid in the development of effective treatment schedules in human patients. SEM micrographs showed the hyperthermal shock and subsequent cell area recovery in an increased resolution at 0 h and 24 h after RF, respectively. SEM micrographs also suggested that the morphology of the cell membrane had altered after RF in malignant cell lines, which may influence the interactions of the cells with their environment (including nano- and microparticles). However, since there are numerous factors that affect a variety of bio-physicochemical processes at the nano-bio interface^16^, we are not able to draw a clear conclusion regarding the influence of the changes in cell surface roughness on the uptake of nano- and microparticles (Fig. 2a,b and S3, Supplementary). Atomic force microscopy images were obtained to provide topographical data of representative cells before and after RF treatment. AFM topographical data suggest that cell retraction primarily is due to a re-organization of the cytoskeleton and cell membrane rather than an expulsion of cellular contents (Fig. 2c1, c2).

Additionally, RF treatment alters the surface roughness of cancer cells. Morphological alterations such as these will undoubtedly influence the initial interactions between cell membrane and therapeutic moieties. Our initial data suggests that when carboxylated quantum dots are incubated with cancer cells which have undergone RF pre-treatment, an increased passive uptake of particles is observed (Fig S5, Supplementary). Further investigation is warranted to establish whether RF pre-treatment can enhance the therapeutic efficiency of nanomedicines, such as gold NP^6^ or gene vectors.

### Cell Motility and Mechanics

The functional relationship between cell motility and cell membrane elastic modulus and adhesion properties was investigated via cell tracking using brightfield and atomic force microscopy (AFM).

### Cell Motility

**Figure 3:**
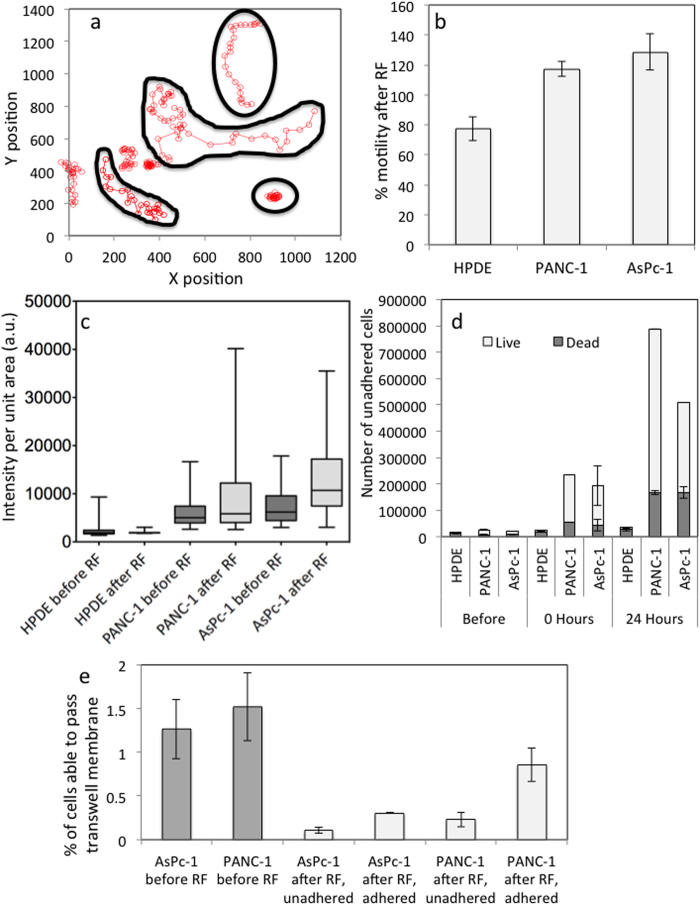
Cell motility in response to RF treatment. **a**) Cell tracking via co-ordinates over 20 h before and after RF. **b**) 2-D motility measured for 20 h before RF treatment and between 4 and 24 h after RF treatment (% motility normalized to motility before RF). **c**) F-Actin expression before and after RF (n = 81 frames), **d**) Cell-substrate unattachment in response to RF treatment, the number of live and dead cells in un-adhered subpopulation after RF treatment and e) 3D migratory behavior of cancer cells before and after a single RF treatment (*p < 0.01).

Cell motility is influential in various pathological processes, such as cancer metastasis, and has therefore generated recent interest^17^,^18^. Cell motility is known to involve continuous mechanosensation^19^, that may be altered by the RF field. Cell motility before and after RF treatment in the sub-population of cells still adhered to the substrate was evaluated in the 2D environment. Motility was measured using ImageJ software by manually selecting the center of mass of a cell and following it through a 20 h time-period by recording its XY co-ordinates at each timepoint (Fig. 3a). These measures revealed that regulation of cell speed and maximum displacement over 20 hours were dependent on RF exposure. Between 4–24 h after RF treatment the PANC-1 and AsPc-1 cells, which remained adhered to the substrate, displayed approximately a 20% increase in maximal displacement, (Fig. 3b). Increases in motility may be due to stress; it is well known cancer cells are more sensitive to hyperthermia^20^ which has been attributed to their dielectric properties^4^. RF treatment caused the motility in HPDE cells to decrease in the 20 hours following RF treatment.

Between 0 and 24 hours after RF exposure, a substantial proportion of malignant cells were seen to detach from the cell culture plate substrate and become suspended in the supernatant. It is well known that when cells undergo necrosis or apoptosis a loss of cell membrane integrity potentiates loss of adhesion and dis-attachment from substrate surfaces. Therefore, the viability of the non-adhered cells found in the supernatant was investigated. The Trypan Blue assay determined that a large percentage of the detached population remained viable after RF treatment (Fig. 3d), which indicates that cells becoming unattached from the substrate is a specific biological response after undergoing RF exposure. The migratory characteristics were investigated in this sub-population via a Boyden Chamber analysis, which found these cells were less able to negotiate a transwell membrane (pore size 5 μm) when compared to untreated cells (Fig. 3e).

### Expression of Tunneling Nanotubes (TNTs)

**Figure 4:**
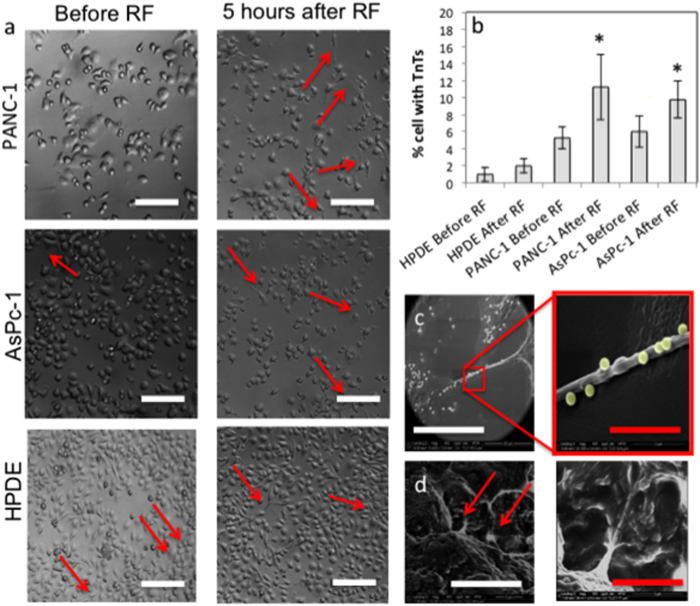
Tunneling nanotubes (TNTs) formation as a result of RF-treatment. **a**) and **b**) RF causes an increase in the formation of Tunneling Nanotubes (TNTs) in malignant cell lines (Red arrows indicate TNTs) (n = 1000, *p < 0.01) (Scale bar = 50 μm). TNTs are thought to be involved in cell-cell signaling and particle trafficking over short-medium distances. **c**) TNTs are also associated with particle trafficking in cancer cell lines, which, to our knowledge has not been documented before. TNTs may provide a mechanism for transfer of dose throughout a malignant population or may offer cancer cells a mechanism where they are able to dilute a toxic dose to ensure survival. (White scale bar = 20 μm, red scale bar = 1 μm). **d**) SEM images of TNTs present in 3D pancreatic tumor environment in spheroid models (White scale bar = 3 μm, red scale bar = 5 μm).

AsPc-1 and PANC-1 displayed a near total retraction of all cellular protrusions, including tunneling nanotubes (TNTs), immediately after RF treatment, which lasted between 0–4 h after exposure. However, overexpression of TNTs (Fig. 4), from the main cell body, occurs from 5 h onwards after RF treatment. TNTs were reported as the means of cell-to-cell interactions, which coordinate the communication between adjacent cells and distant cells in the 3D tissue microenvironment. Although TNTs are present in the healthy cells, pathological conditions (e.g. infectious state, cancer) were previously associated with an increase in the number of TNTs^21^,^22^. TNTs represent an interesting feature of cells, which, generally, is related to the transfer of information between the two neighboring cells. It is possible that the enhanced expression of TNTs in cells, which underwent RF treatment is a reflection of the stress that the cells experience, similar to what was observed by Feratti and colleagues, who describe an overexpression of TNTs when endothelial cells were cultured in serum free media^23^.

Specific changes in cell shape, such as the production of specific cellular protrusions including TNTs and lamelapodia are associated with the re-organization of filamentous actin in the cytoskeleton. Therefore, the F-actin network of malignant and non-malignant cells was stained using phalloidin dye (Fig. 3c). The resolution of the images were limited to 40X magnification during confocal microscopy, as glass plates, which allow a higher magnification, cannot be placed in the RF field. Therefore F-actin organization could not be investigated in detail, however overall expression of F-actin, which was given by the intensity of the phalloidin dye per cell, could be analyzed. RF exposure caused the overexpression of F-actin in malignant cells and the under-expression of F-actin in the non-malignant cells after RF. These molecular changes are in agreement with the changes in motile behavior and overexpression of TNTs that we observed at the cellular length scale.

### Mechanical Analysis

**Figure 5:**
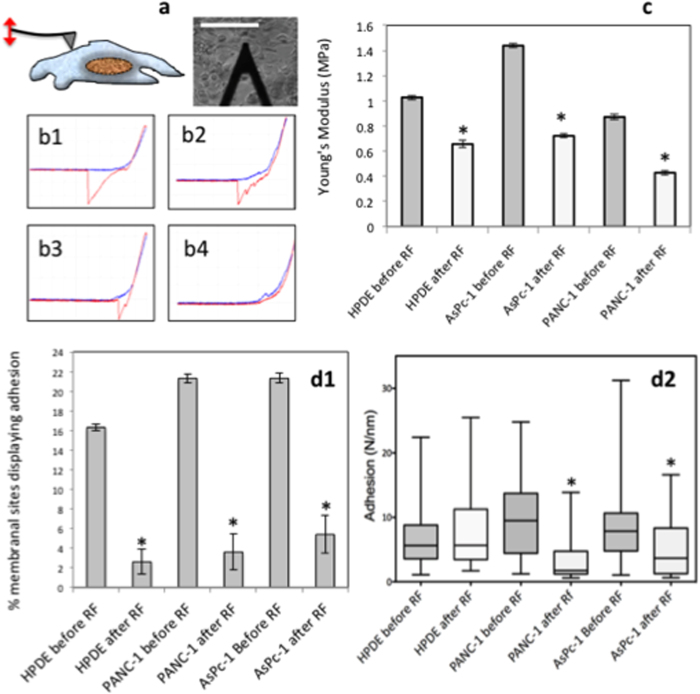
RF affects physical properties of the plasma membrane. **a**) AFM probe aligned with x20 magnification brightfield image guidance, which scans points of interest over cytoplasm (n = 500 points on membrane of 20 cells), (scale bar = 50 μm). **b**) AFM force curves of PANC-1 before RF (B1–B3) and after RF (B4) (Red retraction curve indicates adhesion; blue extension curve indicates elastic moduli). **c**) Elastic Modulus (n = 500 points on membrane of 20 cells). **d**) Percentage of sites which display adhesion on the cell membrane and C) magnitude of adhesion at adhesion sites (n = 500 points on membrane of 20 cells) (*p < 0.01).

AFM allows the high-resolution characterization of the pancreatic cancer cell surface, which provides a platform for the multi-parameter analysis of cell function^24^. For instance, cancer cell mechanics, such as adhesion and elastic modulus, can influence tumor growth and metastatic potential^25^. The elastic moduli decreased in all three cell-lines following RF treatment (Fig. 5), which indicates that the cell membrane had become less stiff and perhaps more fluid in response to RF treatment. Our data show the effect of RF treatment on the physical (mechanical) properties of the whole cells. Although in this manuscript we haven’t examined the effect of RF therapy on the mechanical properties of the nucleus or other organelles, this is one of the aspects that should be addressed in future studies. As an example, Wolf and colleagues have recently shown that cell migration in the confined space was governed in part by the ability of the nucleus to deform under stress^26^.

The number and magnitude of cell membrane adhesion sites across the plasma membrane significantly decreased after RF treatment. This was quantified via the shape of the retraction curve from the force measurements from AFM analysis. *In vivo* cell adhesion is mediated by the interaction of extracellular matrix components with cell-surface molecules, whereas the loss here is a reaction to thermal stress that causes the cell to re-organize its membrane to allow for movement away from the stressor.

### Cell Proliferation

**Figure 6:**
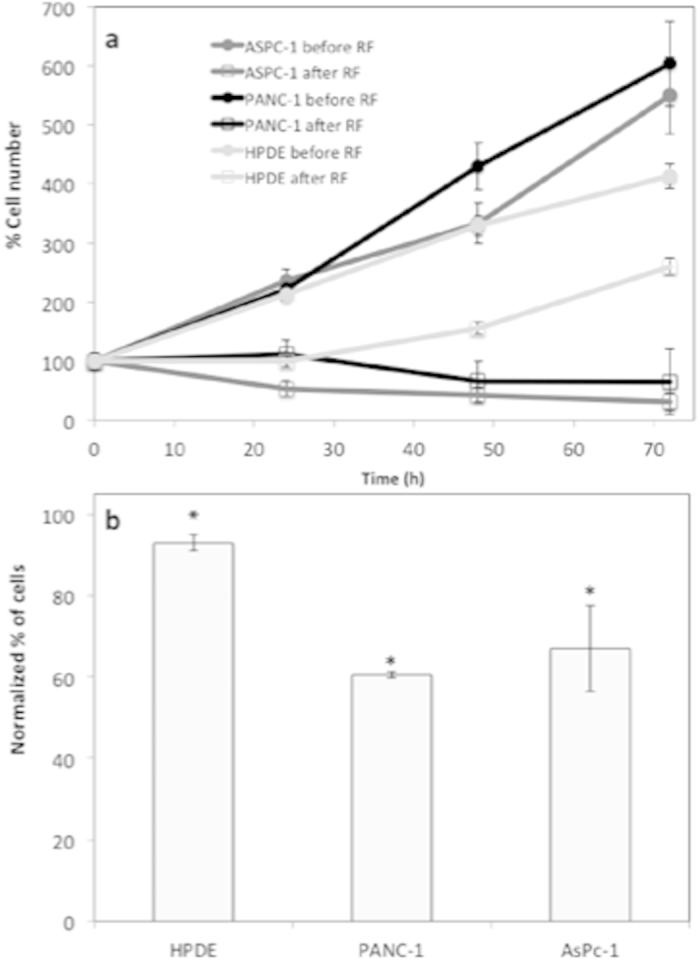
Cell Proliferation in adhered population. WST assay quantified metabolic activity/proliferation in adhered and non-adhered a) AsPc-1, **b**) PANC-1 and **c**) HPDE cells (% proliferation normalized to cell numbers before RF treatment). **d**) Trypan Blue assay quantified the total number of RF treated cells at 48 h. Normalized to the number of cells in untreated plates at the same time point (normalized to number of untreated cells).

Previous studies have shown that the use of noninvasive RF fields decreases viability in human and mammalian pancreatic cancer cells with limited effect on non-malignant cells^3^. The WST assay provides an indirect method for the quantification of proliferative activities within a cell population containing both adhered and non-adhered cells. It must be noted that cell number or metabolic activity cannot be extrapolated as a single measurement from the results of the WST assay. A decrease in absorbance indicates both a decrease in proliferation and/or a decrease in metabolism in PANC-1 and AsPc-1 cell lines (Fig. 6a). HPDE displayed a less drastic effect (Fig. 6a). To obtain a more direct measure of proliferation rate, a cell count at 48 h after RF treatment was carried out using the Trypan blue assay (Fig. 6b). These data suggests that RF disrupts the metabolic activity and proliferation in all cell lines, however HPDE showed a much less drastic response. Additionally, the non-adhered population was singled out for study and were recovered from the supernatant after a single RF treatment and reseeded in fresh media. The non-adhered cells were able to re-establish a cell population, however they also showed decreased proliferation rate between 0 and 1 week after reseeding when compared to untreated cells.

### Cell Response to Multiple RF Treatments

**Figure 7:**
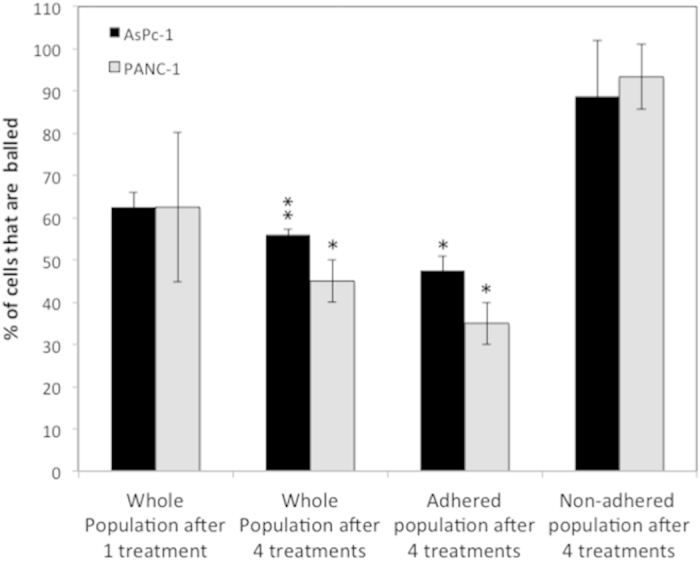
Polymorphic response to RF treatment. Percentage of balled cells in various sub-populations indicates increased thermotolerance and the pre-existence of more heat sensitive cells. (Cytoplasm retracted cell classified as cells, which display at least a 33% decrease in cell area) (*p < 0.01, **p < 0.05).

A patient is likely to receive multiple exposures to RF waves over the course of his/her RF treatment schedule. Therefore it is warranted to investigate the effects of multiple RF treatments on cells. When subjected to two or three RF treatments 24 h apart, both PANC-1 and AsPc-1 cells exhibited cytoplasmic retraction similar to that seen following a single RF treatment. However, after four treatments it was evident that the cytoplasmic retraction in response to the RF treatment declined in the adhered sub-population of cells. This effect, shown in Fig. 7 (Fig. S6, Supplementary) was quantified using a textural analysis algorithm in MATLAB (MATLAB code S7.2, Supplementary), which measured the total area of the space between cells in the monolayer. This area was normalized to the space between cells before RF. The algorithm determined that after a single RF treatment the cell free space increased by 23.7% and after 4 treatments it increased by only 4.2%. The cells found in the supernatant and reseeded in fresh media remained susceptible to thermal shock as nearly 100% of cells in this sub-population were balled after RF treatment (Fig. 7, Figs. S6 and S7.1, Supplementary).

## Discussion

Cancer cell phenotype was altered in response to radiofrequency treatment. Cell morphology and behavior changed which may have consequences for the tumor microenvironment and surrounding tissue architecture. Once a tissue has formed, it remains sensitive to alterations to the shape and mechanics of all its constituents. Cells change their shape when the subtle balance of forces that define their shape is modified — this is analogous to how a small stumble can immediately alter, and can quickly end, a game of tug-of-war. When changes of cell shape and mechanics spread in a tissue, as is the case in cancer, the organization and shape of the entire tissue is necessarily altered^27^. Communications between and among cells are mediated through cell surface receptors and a network of signal transduction reactions. Mechanical forces actively alter large-scale spatial organization of signaling molecules^28^, providing a mechanism for physical forces to directly regulate chemical signal transduction processes. These, in turn, can activate or repress genes, modifying cell and extracellular matrix mechanics, and so on. Therefore, size and morphology are important characteristics of any cell with consequences for tissue, tumor and organ architecture. Malignant cells are often described as having an altered appearance and morphology, which are heterogeneous across a population; cancer pathologists and oncologists routinely use cell and nuclear morphology to stage cancer and propose treatments. Clinical studies have linked tumor cell mass and patient survival to variations in individual cells^29^ and nuclear morphology^30^. Furthermore, the characterization of the cell surface and morphology may indicate the type of interaction and subsequent susceptibility to therapeutics^31^. Knowledge of the source and form of biological variation is an obvious requirement for progressing the understanding of cellular response to chemotherapy. Furthermore, it has previously been shown that in animals tumor blood flow and oxygen delivery is significantly increased when hyperthermia is applied up to 42 °C^32^,^33^ and it been reported that RF heating effects causes vasodilation of capillary networks^34^ that surround solid tumors. In addition to vasodilation, we are observing that 3D tumor spheroids display a 5% decrease in total volume at 0 h and 15% decrease at 24 h after RF treatment (S4a and S4b, Supplementary). This provides an indication that decreases in individual cell area, as seen in monolayer cultures, do indeed play a role in alteration of cell-cell junctions in 3D environments. Also, loss of cell adhesion is observed in 3D tumor spheroids as there are 10% and 40% more non-adhered cells in close proximity to the main spheroid at 0 h and 24 h after treatment, respectively (S4a and S4c, Supplementary). It is currently being investigated if this effect is able to enhance the enhanced permeability and retention (EPR) effect and allow an increased passage of drugs and/or nanovectors through endothelial walls, stromal compartments and tumor cell junctions in solid tumors.

The type of motion, such as linear or circular, is able to give information on the metastatic behavior of cancer cells in addition to the displacement of the cells over a set period of time^2^. The Physical Sciences - Oncology Centers Network of National Cancer Institute^2^ recently investigated the motility characteristics in a 2D environment of non-tumorigenic MCF-10A and metastatic MDA-MB-231 breast epithelial cell lines, which are commonly used as models of cancer metastasis. The MCF-10A cells displayed a circular or pin-wheel style of motility, where their leading edge swung in an arc while the lagging edge often remained pinned in place whereas the more aggressive, MDA-MB-231 cells, moved more linearly, although more slowly^2^. From observation, PANC-1 and AsPc-1 before and after RF did not display any obvious changes in the type of motion although a more clear distinction between them and HPDE cells could be seen both before and after RF. The HPDE cells seemed to occupy the same space and moved around a central point whereas the AsPc-1 and PANC-1 cells did not show any of these restraints. AsPc-1 and PANC-1 displayed increased peripheral motion after RF via the growth of cellular projections, or tunneling nanotubes (TNTs) (Fig. 3b), from the main cell body, which moved in a more aggressive manner in the hours after RF treatment. These unique structures have attracted increasing interest as an under-recognized mechanism of cell-to-cell communication and transfer of cellular contents and have not been fully explored for cancer cell types.

Mechanical changes were also observed in cells in response to RF treatment. The mechanical integrity of cells is regulated by the cytoskeleton, a dynamic and interconnected network of filamentous polymers, regulatory proteins and signaling molecules^35^. The distribution of the actin network plays an important role in determining the mechanical properties of single cells^36^. Alterations of mechanical properties of individual cells can reveal important information about changes in these networks. Studies of a variety of diseases utilizing different experimental techniques have shown that abnormalities in the elastic properties of individual cells are important in tissue homeostasis, cell growth, division and motility^37^ and is also associated with disease pathogenesis and progression^38^,^39^,^40^,^41^,^42^. For example, as cells transform from non-malignant to cancerous phenotype, their cytoskeletal structure changes from an organized to an irregular network, and this change subsequently reduces the stiffness of these cells^25^. Another example is that invasive tumor cells mechanically soften and modify their adhesion to extracellular matrix, which enhances their capacity to escape the primary tumor^42^ and thus measurements of cancer cell stiffness, quantified by the Young’s modulus, have shown a strong correlation between cell deformability and cell malignancy^25^. Alternatively, it may also be indicative of a loss of cell membrane viability and cell health. As previously mentioned any changes in the mechanical properties of the cell membrane will change the interaction with various nanoformulations and thus result in the initial interaction between particle and cell being affected. This may lead to subsequent uptake pathways to be altered. Our initial data indicates that following RF treatment a more diffuse association occurs between PANC-1 and AsPc-1 when dosed with carboxylated quantum dots (Fig. S5, Supplementary) and endocytic pathways are largely by-passed. This may be particularly relevant for introducing drugs or nanoformulations that rely on the avoidance of acidic compartmentalization via the endocytic pathway.

A patient is likely to receive multiple exposures to RF waves over the course of his/her RF treatment schedule. Therefore we investigated the effects of multiple RF treatments on cells. Certain subpopulations of cells displayed evidence of a developed ability to become less susceptible to the RF treatment over time. This was characterized by no change in morphology in response to RF treatment as seen after a single RF treatment. Secondly, multiple RF treatments seemed to ‘sort’ sub-populations by their susceptibility to RF or by their innate behaviors in response to treatment, as there were some cells within the population, which always balled and detached from the substrate and never developed lowered susceptibility. This finding was unexpected and suggests: 1) the preexistence of a subpopulation of cells which are more prone to RF effects and, therefore, more readily become detached from the substrate and/or 2) an emergent subpopulation which displays increased phenotypic plasticity and therefore develop an ability to compensate for the diverse effects of temperature, remain adhered to the substrate surface and survive even after multiple treatments to create an evolutionary bottleneck scenario. This process may protect malignant cells against hyperthermia not only with respect to the maintenance of their reproductive ability but also with respect to general cell metabolism^43^ and thus rendering treatment less effective. Indeed, it has been reported for many years that the sensitivity of cells (and tissues) to hyperthermia is transiently but markedly reduced following an initial heat treatment^44^. This resistance is expressed in cells that survive very mild to severe heat treatments and is due to the upregulation of heat shock proteins^45^. The time required for the development of maximum thermotolerance varies some-what with the magnitude of the initial heat treatment, from a few hours following a very mild initial heat treatment to longer durations following severe initial treatments. This is an important consideration when devising an RF treatment schedule for patients in upcoming clinical trials as thermotolerance due to the development of heat shock proteins has already been reported in many cancer cells lines, including rodent tumor cells following *in vivo* heating^46^. These findings can provide a basis for understanding the mechanisms involved in cancer cell sensitivity to RF as well as the rational design of appropriate schedules of therapy in combination with chemo- or radiation therapy.

In conclusion, our data shows that RF treatment causes significant changes in phenotype of cancer cells, which are different from the reaction of normal cells to RF field. Phenotypical changes occur over several timescales, for instance, cell cytoplasm retraction is an immediate response whereas increases in motility are seen over hours preceding treatment. Furthermore, clear phenotypical differences were observed between cancerous and normal cells in both their untreated states and in their response to RF therapy; malignant cell lines showed a significant increase in intensity per unit area of phalloidin after RF whereas the normal cell line displayed a decrease. Additionally, we have provided some evidence that there are various sub-populations within a whole cancer cell population, which respond differently to RF treatment and also evolve to become somewhat ‘resistant’ to RF therapy after multiple RF treatments occurs within different sub-populations. These changes are reflected in the evolution of observable phenotypical responses over time. The evaluation of phenotype between sub-populations of cells and changes in phenotype in response to RF treatment in 3D cell cultures and *in vivo* is currently being undertaken.

## Methods

### Cell Lines

Two pancreatic cancer cell lines, PANC-1, AsPc-1 were obtained from American Type Culture Collection (ATCC). Cells were maintained in Dulbecco’s modified Eagle’s medium (DMEM) (ATCC) with 10% fetal bovine serum (Invitrogen, USA). Normal human pancreatic ductal epithelial cell line (HPDE) were cultured in keratinocyte serum free media (KSFM) (Invitrogen, USA) and supplemented with bovine pituitary extract and recombinant epidermal growth factor. All cells were cultured in 5% CO_2_ at 37 °C.

### RF treatment of the cells

Cells were seeded at 200,000 cells per well in a 12 well plate and, after 24 hours when reattachment had occurred, were placed in the capacitively coupled RF system, the Kanzius external RF generator. Cells were exposed to RF field (900W, 13.56 MHz) for 5 minutes. For cells undergoing multiple RF treatments, a small amount of media was added every two days but not replaced so that the non-adhered sub-population of cells, which resided in the supernatant, would not be lost. Parameters were measured using brightfield images, which were obtained using ImageXpress (Molecular Devices). No fluorescent probes were used to prevent any artifact in the cell response to the RF fields.

### RF Heating Rate

The RF field was calibrated by temperature, which was recorded in real time via infrared thermography (FLIR Systems Inc., Boston, MA) to ensure a consistent field across the wells within the plate and over time. The temperature increased by 0.32 °C (SD 1.11) within the first minute of exposure and displayed a linear heating rate of 1.38 °C min^–1^ (SD = 0.23) from minute 1 to 5. The final temperature at the 5 minute time point was 38.68 °C (SD = 0.36). These results confirm a similar heating rate in serum containing DMEM and serum free KSFM media (see Supplementary Fig. S8).

### Quantum Dot dosing Schedule

AsPc-1 and PANC-1 cells were seeded at 200,000 cells per well in 12 well plates and after 24 h were treated with RF, as described above. The carboxylated quantum dots (Life Technologies, U.S.A) were vortexed for 5 sec in 18 nM concentration in 2 mL of cell media and added to cells, which had and had not undergone RF pre-treatment. After 2 h incubation at 37 °C, the media containing the quantum dots was replaced with 4% paraformaldehyde for 15 mins to fix the cells. The cells were then washed gently with PBS and incubated for 10 mins with DAPI nuclear stain (Life Technologies, U.S.A) and the PBS was added to the well. Confocal microscopy was performed where cells were imaged at 20x magnification in three channels: the DAPI and the carboxylated quantum dots in fluorescence mode with excitation and emission wavelengths of 350/460 nm and 350/655 nm respectively. Quantification of the number of quantum dots in the peri-nuclear region relied on a mask being drawn around the perimeter of the DAPI stained nucleus and the mean intensity of the quantum dots measured.

## Evaluation of physical characteristics of the cells

### Analysis of cell area

To determine the 2D cell area, PANC-1, AsPc-1 and HPDE were grown to approximately 70% confluency and brightfield live cell images were taken of the same cell populations before and at every hour for 24 hours after RF treatment. Manual segmentation of the brightfield images at time-points before, 0 and 2 h after RF exposure was performed by highlighting the cell perimeter using ImageJ 1.47 Software (National Institutes of Health) and measuring the area within the selection. Over 600 cells were measured for statistically robust results that considered a large percentage of the whole cell population.

### Assessment of cell motility

2-D motility data was obtained by tracking the position of single cells (n = 50 per group) within a field of view by manually selecting the center of each cell and recording the XY co-ordinates 20 h before RF and at time-points 4–24 h after RF using ImageJ 1.47 Software (National Institutes of Health). Measurements were taken from the same fields of view and in the same cell populations before and after RF. The distance that each cell had travelled within the time period was then plotted from the co-ordinates. Time-points 0–4 h after RF was not considered, as cells were balled and non-motile.

### Transwell Cell Migration Assay

AsPc-1 and PANC-1 cells were seeded at 200,000 cells per well in a 12 well plates and after 24 h were treated with RF, as described above. Immediately after RF treatment, the detached (floating in the media) cells were collected with the supernatant and the attached cells were further detached from the surface by trypsinization. Ten thousand cells from each group were resuspended in serum free base media and placed in 12 well Transwell plates (Corning, Sigma-Aldrich, USA) with a pore diameter of 5.0 μm. Media containing 20% fetal bovine serum was used as the chemo-attractant. After 4 days, the number of cells that had passed the transwell membrane and attached to the bottom of the plate were counted using DAPI staining and brightfield microscopy.

### Atomic Force Microscopy

Topographical quantification of cell shape and cell membrane adhesion and elastic moduli data was obtained via atomic force microscopy. Briefly, cells were grown on a plastic cover slip at a confluency of 70%, which was placed in the 12 well plates for RF treatment. At various time-points after RF treatment the media was carefully removed and cells were washed twice with HBSS which was pre-warmed to 37 °C. The cells were then incubated for 10 min with 3% Glutaraldehyde on a rocking table at room temperature. The cells were then washed thrice with de-ionized water and placed in pre-warmed HBSS for AFM imaging. For force-indentation analysis the AFM used a conical tip with a radius of 3 nm and a spring constant of 0.06124 N/m and a load force of 2 nN and a ramp size of 2 μm. The AFM tip scanned 100 points on the cytoplasm regions of 5 cells per group, which obtained 500 force curves per group. The resulting force-indentation curves were fitted to a Sneddon conical model in 100 nm intervals over the cytoplasm region of the cells to yield an elastic moduli distribution of cells, which had undergone RF treatment, and cells, which had not. Adhesion curves were analyzed by measuring the axis displacement from the point the cell separates from the tip to the base line via manual means.

### Evaluation of gaps between cells following RF treatment

For the quantification of inter-cellular space within the cell monolayer during multiple RF treatments an image analysis algorithm was created using MATLAB programming software (R2011a). The algorithm featured texture range filtering and used the morphological pixel dilation and erosion function to determine the maximum and minimum values in a specified pixel neighborhood (3 × 3 pixels). A region occupied by a cell generally causes the pixel intensity range within this neighborhood to be higher than that of a region where no cells reside and therefore is able to create an output image containing black pixels indicating space and white pixels indicating an area occupied by cells. Percentage space was calculated by the number of black pixels divided by the number of black and white pixels multiplied by 100. (S6.2, Supplementary).

### Scanning Electron Microscopy (SEM)

For *SEM* imaging, cells were grown to an approximate confluency of 70% on a plastic cover slip in a 12-well plate. After RF exposure cells were fixed at various time-points by washing thrice with 0.1 M Sodium Cacodylate Buffer (*CDB)* and bathed in 2.5% Glutaraldehyde for 15 minutes at room temperature before being carefully washed thrice in 0.1 M CDB and subjected to an ethanol series for dehydration. Finally, the cells were incubated in 50% T-Butanol and 50% Ethanol for 5 minutes and mounted on an *SEM* stub in readiness for imaging. Immediately before imaging the samples were sputter coated with 50% Platinum 50% Palladium at a thickness of 4 nm ± 0.2 nm to ensure conductivity.

### Evaluation of Actin expression

The cytoskeleton was stained with flourochrome-conjugated phalloidin to quantify actin expression in response to RF treatment. Firstly, untreated cells and cells which had undergone RF treatment were fixed. PBS was removed and cells were washed thrice with PBS before being incubated with Triton X100 (0.1%) for 5 min for permeabilisation. Cells were then washed thrice with PBS and Alexa-Fluor 546 phalloidin (Life Technologies, USA) was added for 30 mins. Phalloidin stain was removed and cells were washed thrice with PBS before being re-suspended in PBS for imaging. All steps during the staining process were conducted at room temperature in a dark room. Since, the structural characteristics of the actin network, namely the orientation parameter, could not be analyzed as cells grown on optical glass plates/slides cannot be subjected to the RF field and plastic has a limit of 40X resolution due to its inferior refractive properties. Therefore, results considered total expression of F-actin, which was measured by the pixel intensity of over 5,000 cells.

### Assessment of cell proliferation

WST-1 reagent was used for quantification of cell proliferation and metabolic activity before and at various time points after RF treatment. 200,000 cells were seeded per well in 2 mL media in a Costar 12 well plate. After 24 h the cells were exposed to RF treatment. The supernatant media, containing the non-adhered subpopulation of cells, was removed and placed in a 5 mL ependorf. The adhered cells were exposed to trypsin for 5 min until total unattachment had occurred. Two mL of media was added to the plate and then both sub-populations of cells were mixed and centrifuged for 5 mins at 300 xg. Two mL of fresh media were added and the cell suspension was mixed via pipette action. 100 μL of cell suspension was then added to a 96 well plate for dual wavelength analysis (450 and 600 nm) on a BioTek Synergy H4 hybrid multi-mode microplate reader. Results were reported in absorbance units. This procedure was repeated at time points before and at 0, 24 and 48 h after RF. As a control WST-1 absorbency was measured at 0, 24 and 48 h after seeding in cell populations that had not undergone RF treatment.

The trypan blue assay was carried out to measure the number and viability of cells present in the supernatant before and after RF treatment. At various time points after RF exposure, 2 mL of supernatant was removed from each well and centrifuged for 5 min at 300 xg. After centrifuging, the supernatant was carefully removed and 500 μL of fresh media was added to the ependorf. The cell suspension was mixed via pipette action, and 10 μL of cell suspension and 10 microL of Trypan blue stain was thoroughly mixed together in a 1 mL ependorf before being pipetted onto a cover slide in readiness for cell counting using the Cell Countess (Life Technologies, USA).

## Additional Information

**How to cite this article**: Ware, M. J. *et al*. Radiofrequency treatment alters cancer cell phenotype. *Sci. Rep*. **5**, 12083; doi: 10.1038/srep12083 (2015).

## Supplementary Material

Supplementary Information

## Figures and Tables

**Table 1:** Cell physical parameters, methods and measurements.

Cell Physical Parameter	Method	Measurement
Morphology		
Cell shape and size	Manual segmentation of brightfield images	Cell area
	Scanning Electron Microscopy	Visual characterization of cell shape
	Atomic Force Microscopy	Topographical quantification of cell shape
Porosity	Scanning Electron Microscopy	Visual analysis of pore formation
Motility and mechanics		
Cell motility	Manual segmentation of brightfield images	Distance travelled in 24 h before and 24 h after RF treatment
Elastic Modulus	Atomic Force Microscopy	Cell membrane stiffness
Cell detachment	Trypan blue assay	Number of cells in supernatant
Cell adhesion	Atomic Force Microscopy	Number and degree of adhesive sites on the cell membrane
Cell Proliferation	Manual count of brightfield images and Trypan blue assay	Number of cells adhered to substrate Number of cells in the supernatant
