# Supplementary material for: Radiofrequency treatment alters cancer cell phenotype
Source: Sci Rep. 2015 Jul 13;5:12083. doi: 10.1038/srep12083 (PMC4499808; doi:10.1038/srep12083)
Supplement: Supplementary Information [file srep12083-s1.pdf]

Radiofrequency treatment alters cancer cell phenotype

Matthew J. Ware[1,2], Sophia Tinger[1], Kevin L. Colbert Jr.[1],Stuart J. Corr[3], Paul Rees[2], Nadezhda Koshkina[4], Steven Curley[3], H.D.Summers[2] and Biana Godin[1]*

[1] Department of Nanomedicine, Houston Methodist Research Institute, Houston, Texas, USA
[2] Centre for Nanohealth, College of Engineering, Swansea University, Swansea, UK
[3] Baylor College of Medicine, Houston, Texas, USA
[4] MD Anderson Cancer Centre, Houston, Texas, USA
*Corresponding author (bgodin@houstonmethodist.org)

Supplementary Data 1

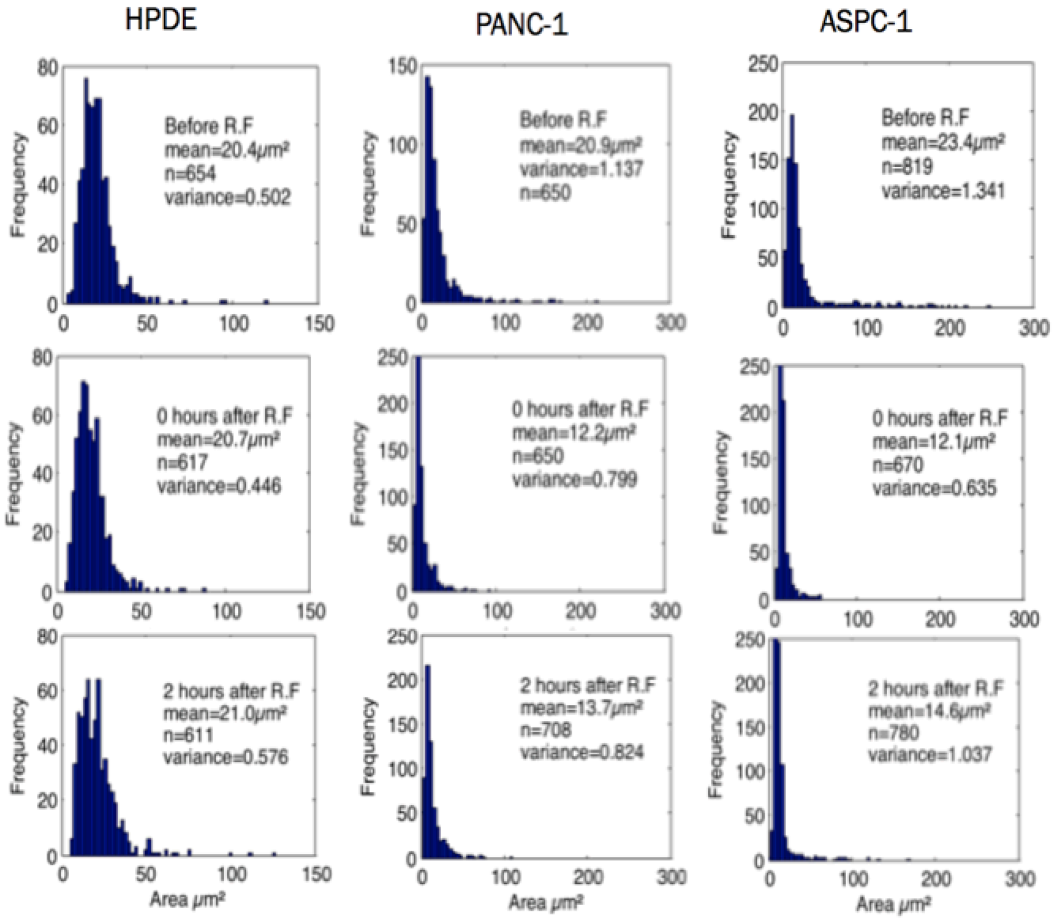

Figure S1: cell cytoplasm retraction 0 and 2h after RF in HPDE, PANC-1 and AsPc-1 cell lines.

Supplementary Data 2

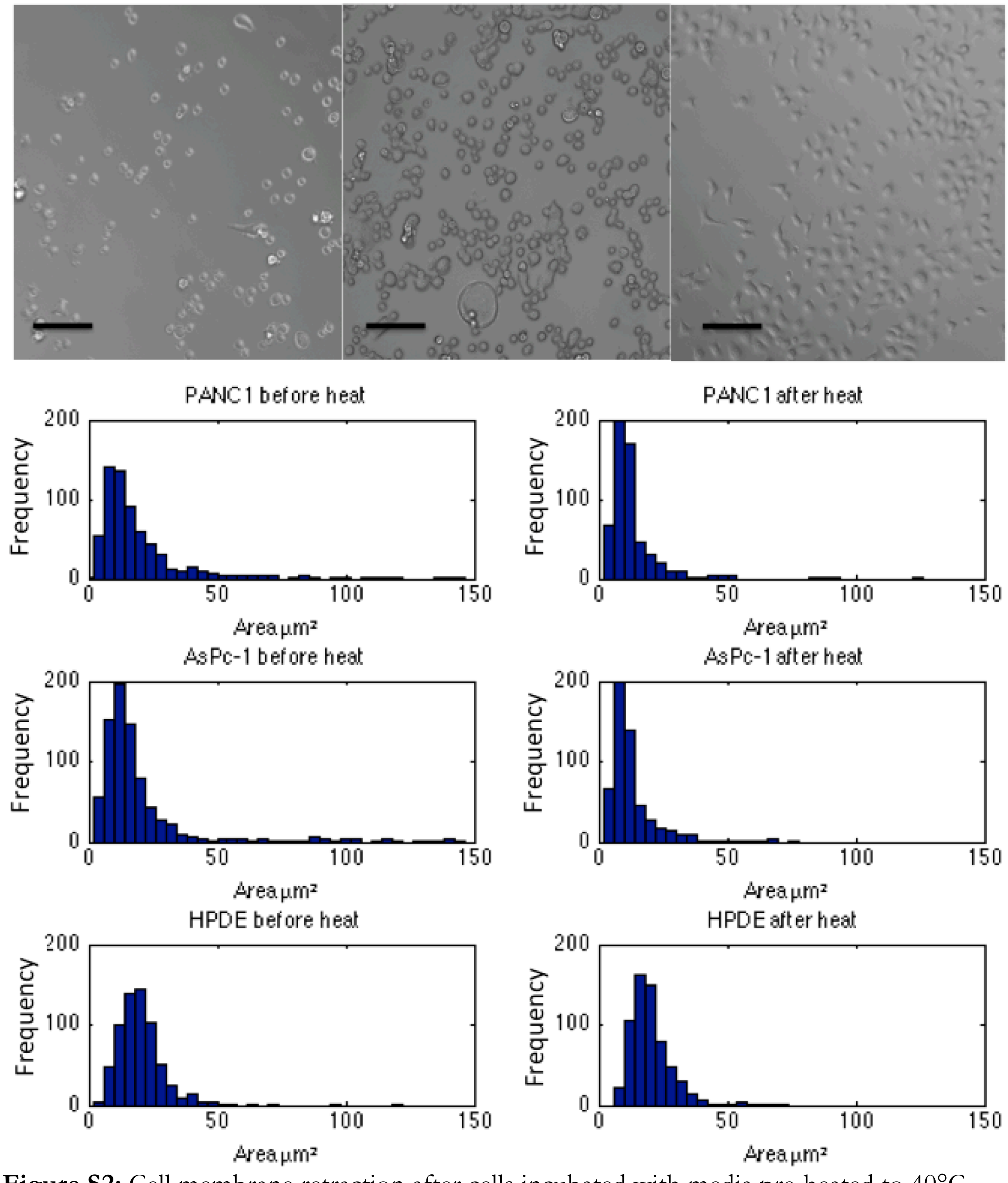

Figure S2: Cell membrane retraction after cells incubated with media pre-heated to 40°C. The same membrane retraction is seen in cells incubated with 40°C pre-heated media, which indicates membrane retraction is a thermal shock. Top) Brightfield images of PANC-1 (left), AsPc-1 (middle) and HPDE (right) (Scale bars = 100µm), Bottom) cell area before and after incubation with 40°C pre-heated media.

Supplementary Data 3

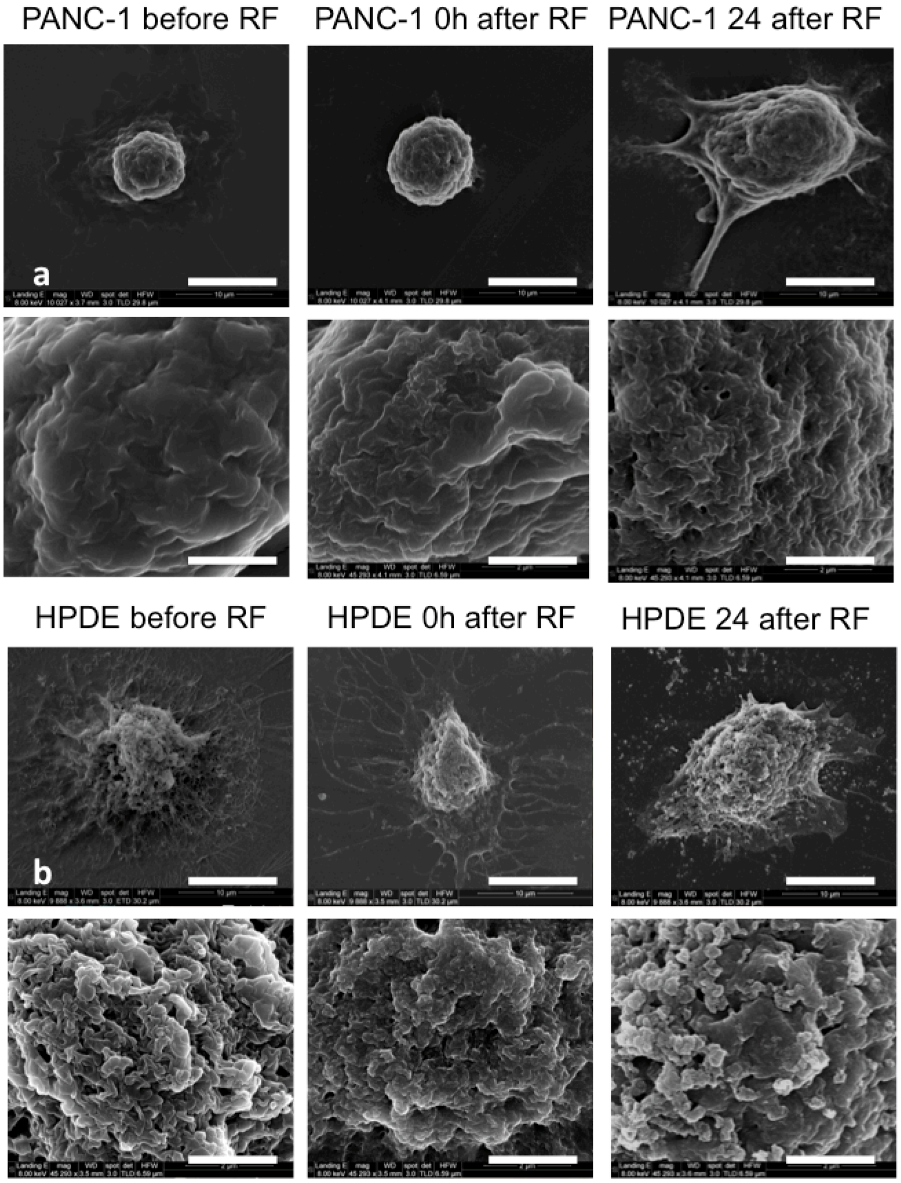

Figure S3: Cell level analysis of RF response. Cell hyperthermal shock indicated by cytoplasm retraction and recovery in malignant cell lines. a) Scanning electron micrographs of PANC-1 cells at times points before, 0 and 24h after RF exposure (top row 10027x magnification, scale bar 10µm, second row from top 45293X magnification, scale bar 2 µm). b) Scanning electron micrographs of HPDE-1 cells at times points before, 0 and 24h after RF exposure (top row 10027x magnification, scale bar 10µm, second row from top 45293X magnification, scale bar 2 µm).

Supplementary Data 4

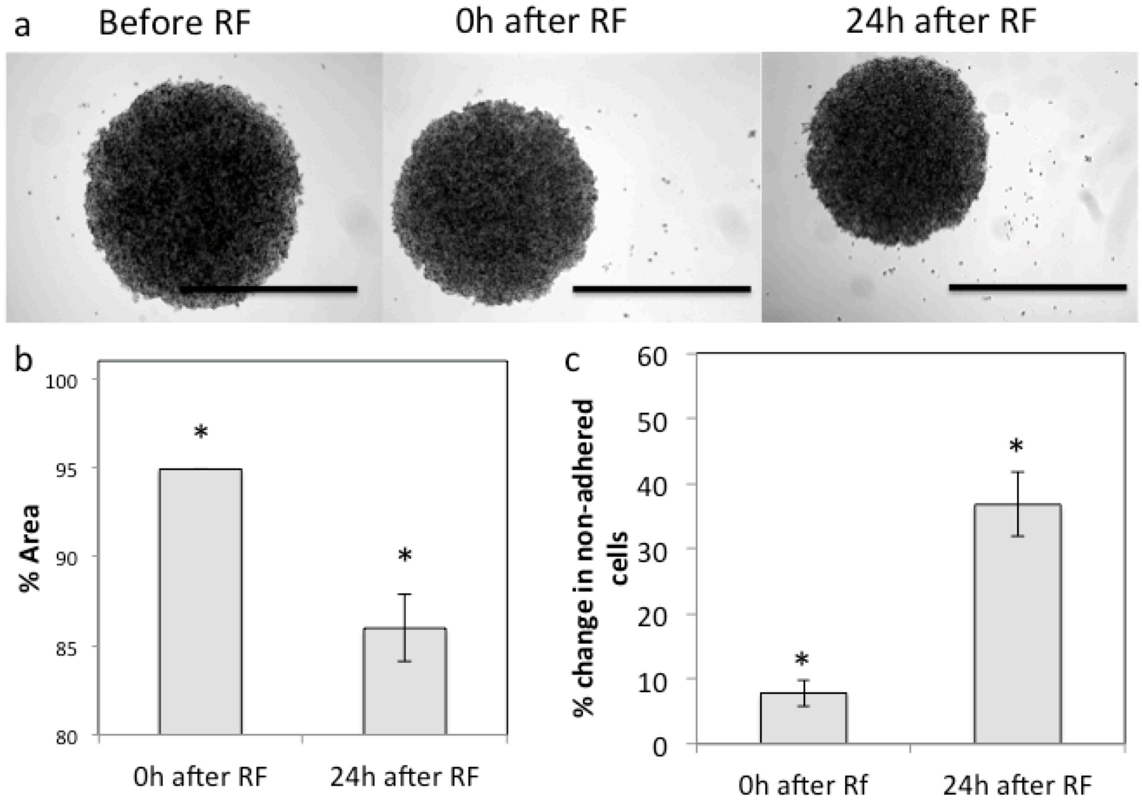

Figure S4: Spheroid area before and after RF treatment. 3D tumor spheroids display a 5% decrease in total volume at 0h and 15% decrease at 24h after RF treatment. a) A 3D tumor spheroid before and at 0h and 24h after treatment (Scale bar =1000μm), b) 3D tumor spheroids decrease in cell area after RF treatment (normalized to the area before RF) and c) Number of detached cells in close proximity to the 3D tumor spheroid at 0h and 24h after RF treatment (normalized to the number of non-adhered cells before RF) (n=25 spheroids) (*p<0.01).

Supplementary Data 5

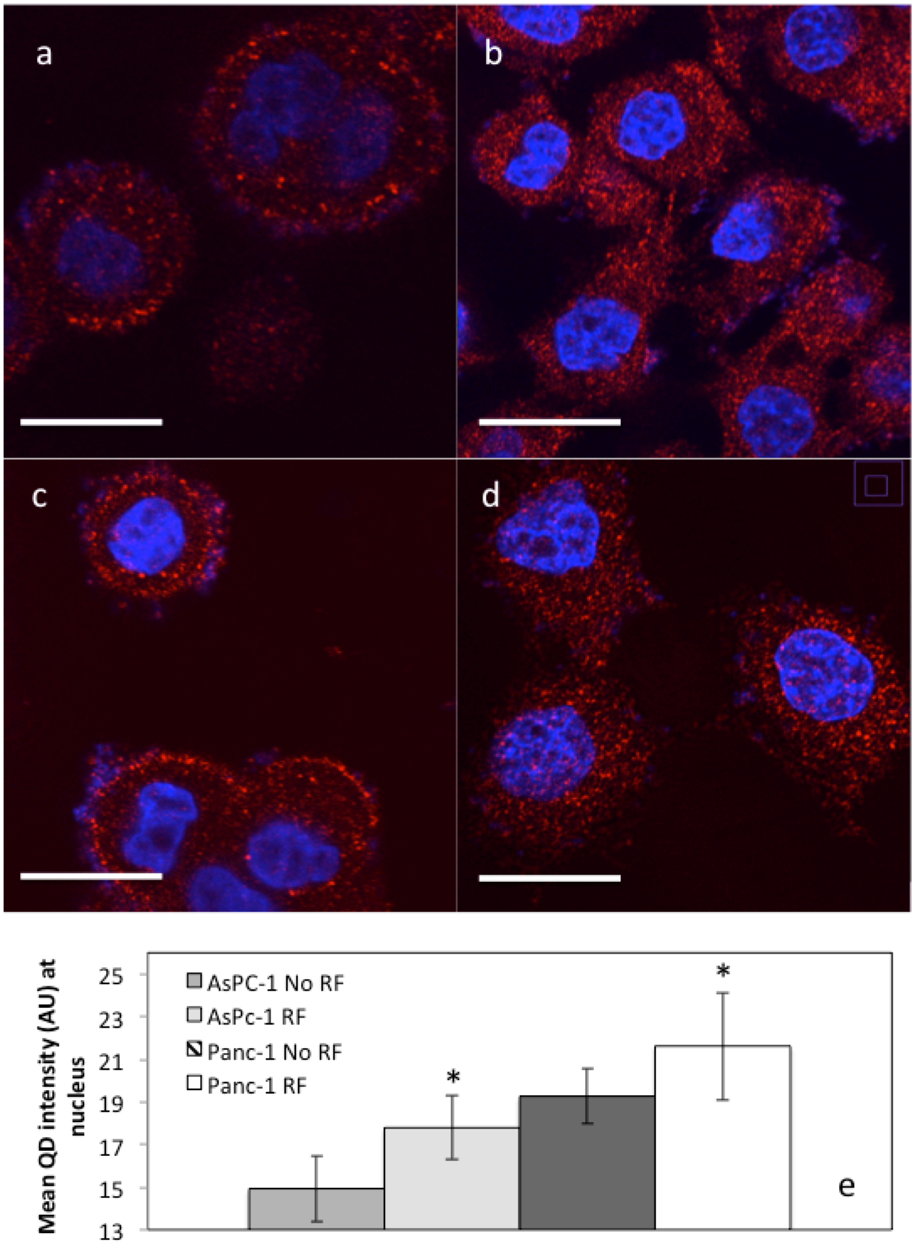

Figure S5: Confocal microscopy of PANC-1 and AsPc-1 cells incubated with carboxylated quantum dots for 2h with and without RF pre-treatment. Confocal images (40X) of AsPc-1 cells a) before RF treatment and b) 0h after RF treatment and of PANC-1 cells c) before RF treatment and d) 0h after RF treatment. e) the mean QD intensity at nucleus. (Scale bar =15 µm) (n=25 cells) (*p<0.01).

Supplementary Data 6

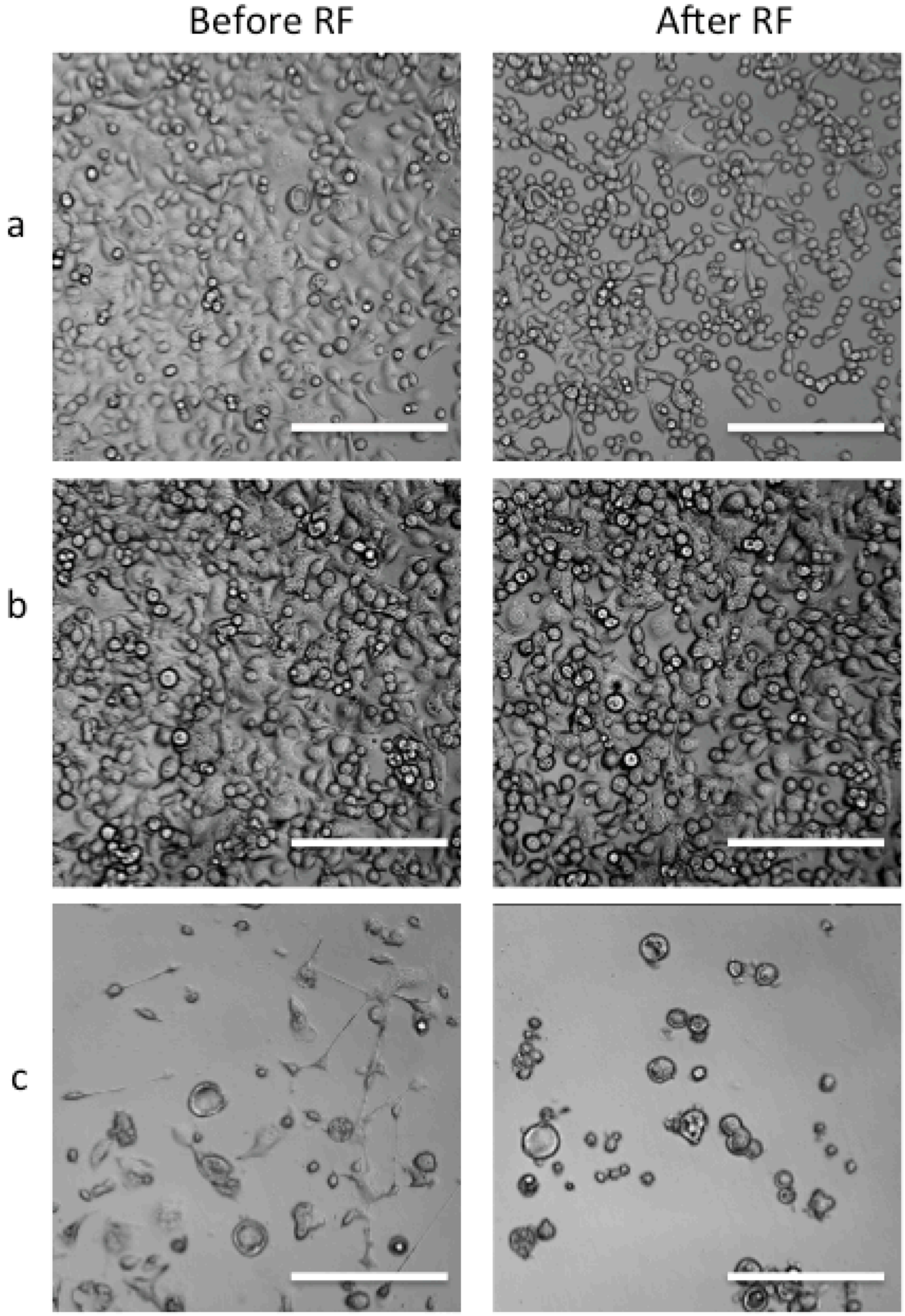

Figure S6: Multiple RF treatment cell response. a) AsPc-1 cells before and 0h after a single RF treatment. b) AsPc-1 cells before and 0h after 4 RF treatments. c) AsPc-1 cells before and 0h after RF treatment. (Scale bar 100 μm).

Supplementary Data 7

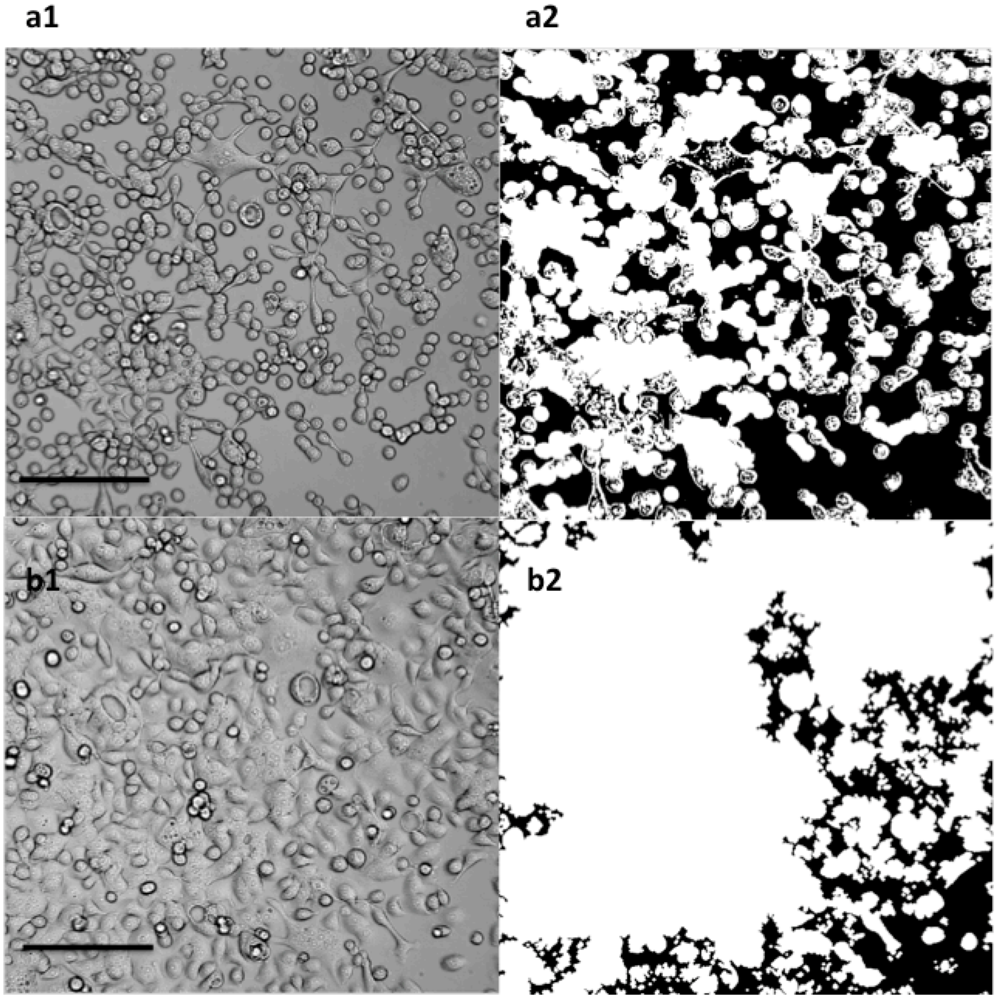

Figure S7.1: Textural algorithm MATLAB code approximates the 'space' between cells before and after RF from brightfield images. a1 and b1) brightfield images of PANC-1 cells after single and multiple RF treatments respectively, and a2 and b2) binary images of a1 and b1 from MATLAB textural algorithm code. (Scale bar 100 μm).

Supplementary Data 7, continued

S7.2: MATLAB Textural algorithm code

```
clear;
cd('RF images before_after_RF');% specifies the directory or folder the
image is located
%Before RF image
imgbefore1=imread('PANC-1 before 1RF site 35.png'); %imports the image
by image name
imgbefore2 = imresize(imgbefore1, 1); %resizes the original image
(img1) by making it 25% smaller
%after RF image
imgafter1=imread('PANC-1 after 1 RF site 35.png'); %imports the image
by image name
imgafter2 = imresize(imgafter1, 1); %resizes the original image (img1)
by making it 25% smaller
%Displaying Brightfield images
figure(1),imshow(imgbefore2),title('Before RF Brightfield');
figure(2),imshow(imgafter2),title('After RF Brightfield');
%texture analysis before Rf
beforefilt = rangefilt (imgbefore1,ones(3));
imgbefore3=im2bw(beforefilt, graythresh(beforefilt));
imgbefore5=imfill(imgbefore3,'holes');
imgbefore6 = imopen(imgbefore5, ones(1,1));
sebefore = strel('diamond',5);
imgbefore7=imdilate(imgbefore6,sebefore);
imgbefore8=imfill(imgbefore7,'holes');
figure(3),imshow(imgbefore8),title('Before RF + image processing');
%texture analysis after Rf
afterfilt = rangefilt (imgafter1,ones(3));
imgafter3=im2bw(afterfilt, graythresh(afterfilt));
imgafter5=imfill(imgafter3,'holes');
imgafter6 = imopen(imgafter5, ones(1,1));
seafter = strel('diamond',1);
imgafter7=imdilate(imgafter6,seafter);
%imgafter8=imfill(imgafter7,'holes');
figure(4), imshow(imgafter7),title('After RF + image processing');
%Calculating percentage of gap change
sumOfAllGrayLevelsbefore = sum(imgbefore7);
mbefore=mean(sumOfAllGrayLevelsbefore)
sumOfAllGrayLevelsafter = sum(imgafter7);
mafter=mean(sumOfAllGrayLevelsafter)
Percentage_difference_of_gap_space=100-((mafter./mbefore).*100)
```

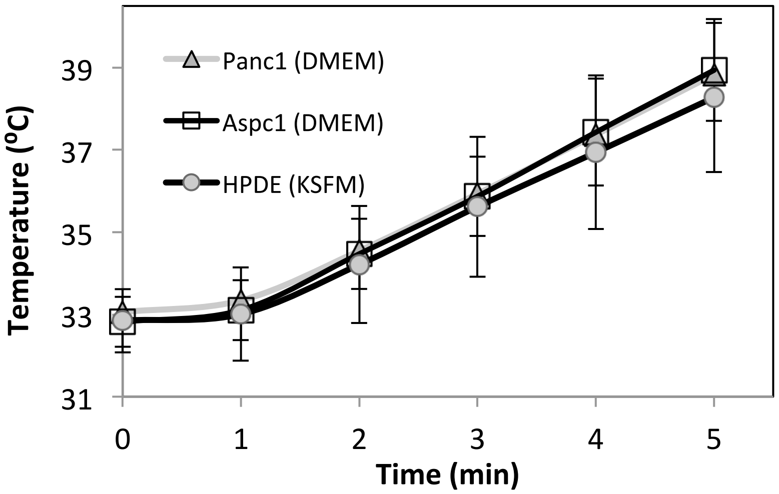

Figure S8: Heating rate. The RF field was calibrated by temperature that was recorded in real time via infrared thermography to ensure a consistent field across all wells within a 12 well plate.
